# Is there silicon in flowers and what does it tell us?

**DOI:** 10.1002/ece3.10630

**Published:** 2023-10-17

**Authors:** Jonas Schoelynck, Petra De Block, Eva Van Dyck, Julia Cooke

**Affiliations:** ^1^ Department of Biology, ECOSPHERE Research Group University of Antwerp Wilrijk Belgium; ^2^ Meise Botanic Garden Meise Belgium; ^3^ Earth, Environment and Ecosystem Sciences The Open University Milton Keynes UK

**Keywords:** angiosperms, biogenic silica, evolution, pollination

## Abstract

The emergence of flowers marked an important development in plant evolution. Flowers in many species evolved to attract animal pollinators to increase fertilisation chances. In leaves, silicon (Si) discourages herbivores, for example by wearing down mouthparts. Flowers are essentially modified leaves and hence may also have the capacity to accumulate Si. If Si in flowers discourages animal visitors as it does in leaves, Si accumulation may be disadvantageous for pollination. Whether flowers accumulate Si, and what the implications may be, was not known for many species. We analysed leaves and flowers of different taxa, separated into their different anatomical parts. Flowers mostly have low Si concentrations in all parts (mean ± SE of BSi in mg g^−1^ was 0.22 ± 0.04 in petals, 0.59 ± 0.24 in sepals, 0.14 ± 0.03 in stamens, 0.15 ± 0.04 in styles and stigmas and 0.37 ± 0.19 in ovaries for a subset of 56 species). In most cases, less Si was accumulated in flowers than in leaves (mean ± SE of BSi in mg g^−1^ was 1.51 ± 0.55 in whole flowers vs. 2.97 ± 0.57 in leaves in 104 species) though intriguing exceptions are found, with some species accumulating more Si in flowers than leaves. The large variation in concentration among flowers across the taxa examined, with a particularly high concentration in grass inflorescences, tantalisingly suggests differences in the use of Si for flowers across plant groups. We conclude that the study of the functions of Si for flowers warrants more attention, with pollination strategy a potential contributing factor.

## Graphical abstract

Whether flowers accumulate Si and what the implications may be was largely unknown. We analysed the flowers separated into their different anatomical parts, and leaves of different taxa. We conclude that flowers contain Si, in large amounts for some species, and hence the function of this element warrants more attention.

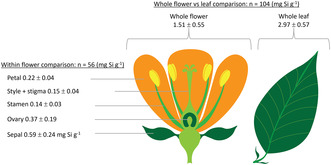

## 1 INTRODUCTION

Plants accumulate biogenic silicon (BSi) in concentrations between 0.1% and 10% of plant dry weight (Epstein, 1999). Most of the absorbed Si is stored as microscopic amorphous Si structures in the plant (including phytoliths). The amorphous Si structures are produced both intra‐ and extracellularly (Hunt et al., 2008; Nawaz et al., 2019). They occur in cell walls, cell lumina and intercellular spaces (Prychid et al., 2003; Richmond & Sussman, 2003), and the Si structures formed and their location in the plant tissue vary widely between plant families (Currie & Perry, 2007). Si is not considered an essential element for plants, but it does have a range of positive influences on overall plant fitness (Ma et al., 2011; Raven, 2003). Si provides, for instance, better resistance to pathogens and higher tolerance to drought (Richmond & Sussman, 2003), structural rigidity (Schoelynck et al., 2010), relief from the negative effects of heavy metals (Song et al., 2014), and increased growth and productivity of agricultural crops in stressful conditions (Ma et al., 2001).

Deposits of BSi in leaves can deter or reduce herbivory, through deposition in the leaf epidermis, or in hairs, trichomes and spines (Hartley & DeGabriel, 2016; Reynolds et al., 2009). For insects, BSi can wear down mouthparts of phytophagous larvae (Massey & Hartley, 2009) and some lepidoptera preferentially select leaves with lower BSi to lay their eggs on (Correa et al., 2005). It is less clear whether BSi causes tooth damage in mammals (see Strömberg et al., 2018 for discussion), but increased chewing and other negative impacts are demonstrated (Johnson et al., 2021). In a range of insect and mammalian chewing herbivores, BSi can reduce the amount of nitrogen an animal can extract from leaves (Hunt et al., 2008; Massey & Hartley, 2006), inhibit microbial digestion (Harbers et al., 1981) and induce other defence mechanisms (e.g. Fauteux et al., 2006). Impacts are negligible or less clear for sucking insects (Johnson et al., 2021). The ability to wear down the mouthparts of insect herbivores and cause poorer digestibility makes silicon‐rich plants a less attractive food source for both insects and mammals (Reynolds et al., 2009; Shewmaker et al., 1989). Silicon uptake can be induced by herbivory (Hartley & DeGabriel, 2016). Induced BSi accumulation in grass leaves has been purported to drive population cycles in voles (Massey et al., 2008), but the induced nature of this defence also suggests there are costs involved in its deployment (Karban & Myers, 1989).

The uptake of Si by vascular plants is a complex process characterised by selective transport and accumulation in different tissues, which differs between and within plant species (Pontigo et al., 2015). Si uptake by plant roots involves two processes: radial transport of Si from an external solution to the cortical cells and the release of Si from the cortical cells to the xylem (Mitani & Ma, 2005). The uptake can be passive (along with water uptake), or by means of special transporters that actively take up Si (Mitani & Ma, 2005). From the cortical cells, Si is then released into the xylem. This is again not the same for all plant species, and the density of transporters in the epidermis of the roots, together with the presence or absence of transporters to the xylem, are important factors determining the amount of Si in the different plant parts (Mitani & Ma, 2005; Strömberg et al., 2018). At least four types of Si transporters have been identified so far which are involved in the uptake and transport of Si throughout the plant, and homologues of these have been found in several flowering plant species (reviewed in Mandlik et al., 2020). From the xylem, Si moves along the transpiration system; it is concentrated by the loss of water during the transpiration process and is converted to amorphous SiO_2_ structures, often referred to as BSi. Much research into plant BSi has therefore focused on leaves, which are perceived to accumulate the most Si among plant parts (Pontigo et al., 2015). There are studies of Si in other plant parts, including roots (e.g. Maguire et al., 2017), wood and bark (e.g. Clymans et al., 2016), and flowers (e.g. Nakamura et al., 2021; Parry & Hodson, 1982), but in comparatively few species and for less functions than for leaves. One plant part that has received very little attention is flowers, where research has focussed on grasses and cereals, and little is known about the accumulation and functions for the many other plant groups.

Flowers are unique to Angiosperms. They are essentially modified leaves on a shortened floral axis, with very short internodes, and no axillary buds (Stevens et al., 2019). Flowers vary enormously between species in size, colour, texture and shape (Harder & Barrett, 2006). There is debate as to why flowers are so diverse within Angiosperms. It is generally accepted that diversity in animal pollinators is mainly responsible for this, in combination with other processes such as predation pressure and the abiotic environment of the plant (Galen, 1999). Within the Angiosperms, there are three main modes of pollination: animals, wind or water. Combinations of wind and animal pollination also occur (Culley et al., 2002). The vast majority of Angiosperms are pollinated by insects and other animals (Ollerton et al., 2011). Having flowers and fruits gives Angiosperms certain advantages over Gymnosperms because they attract animal pollinators and seed dispersers, increasing the fertilisation and reproduction chances. Wind pollination in Angiosperms probably evolved from insect pollination at times when pollinators were insufficient (Friedman & Barrett, 2009). The structure of flowers is highly variable; sometimes, some organs may be fused with each other. It is also possible that some organs have been lost during evolution. For example, unisexual flowers lack (functional) organs of the opposite sex. The structure and appearance of flowers are often linked to the mode of pollination. For instance, flowers of wind‐pollinated plants often have reduced perianth parts, are odourless, less brightly coloured, unisexual and nectarless. In flowers pollinated by animals, it is the opposite (Friedman & Barrett, 2009) with bird‐pollinated flowers often red and insect‐pollinated flowers often blue, yellow or white, resulting from pollinator‐mediated selection driven by the colour perception capacity of these animal groups (Trunschke et al., 2021). Flower longevity also differs between species (from only a few hours to several weeks) and individuals (Ashman & Schoen, 1994; Primack, 1985). Variation in flower longevity occurs between species in the same habitat or species with different pollination systems, but also between members of the same species and male and female flowers of the same species (Primack, 1985).

Angiosperm flowers have thus evolved to attract animals (pollinators), whereas BSi in leaves discourages animals. These two statements seem contradictory given that flowers are essentially modified leaves. This leads to the hypotheses that flower [BSi] will be lower than leaf [BSi] in the same species and that evolution selects for lowered flower [BSi] or possibly complete exclusion. To gain more insight, we analysed flowers and leaves of diverse plant species. The following three research questions were addressed:

1. Is BSi in flowers common and are there differences in [BSi] between different flower parts across the angiosperms?
2. How does BSi concentration in flowers relate to [BSi] in leaves of the same species across the angiosperms?
3. What can we learn from aligning [BSi] in flowers to phylogenetic evolution of flowering plants?

## 2 MATERIALS AND METHODS

Plant materials were collected from Meise Botanic Garden, Belgium, both from greenhouse and outdoor collections, between 19 February 2019 and 12 May 2020 (see Tables S1 and S2 for species lists). Species were randomly selected when flowering and covered a wide phylogenetic variation (grouped as basal Angiosperms, Magnoliids, Chloranthales, Monocots and Eudicots). Cultivars were avoided. Details of the plant species were retrieved from the Living Plant Collection Database (Living Plant Collections Database (LIVCOL), 2019), and the naming and classification were checked and supplemented with recent information if needed.

Flowers (including sepals) and leaves were removed by hand from selected species. Sampled flowers were always fully open. Sampled leaves were mature and visually healthy. Leaf and flower samples came from the same individual. To address the first research question, to find out whether different flower parts contained the same amount of Si, flowers of 56 plant species were collected for dissection into their different organs. Subsequently, to address the second and third research question, 104 species were sampled for flowers (whole) and leaves. Forty‐two of these were herbaceous species and 62 were woody. Thirty‐three plant species were common to both datasets. Though we tried to sample for a broad phylogenetic coverage of the Angiosperms, we were restricted by flower size and seasonal presence, and historical species selection for showy flowers in the botanic garden. This may have skewed the dataset towards larger and easy‐to‐dissect flowers.

Dissection was done immediately after collection. Care was taken to gently brush any pollen away from other organs to avoid contamination. Samples of leaves and whole flowers, and of dissected organs (sepal, petal, style + stigma, ovary and stamen), were dried at 70°C for 48 h and ground to powder. BSi was extracted from ~30 mg of sample, using an alkaline extraction method in 0.5 M NaOH for 5 h at 80°C (DeMaster, 1981). Extractions were filtered over 0.45 μm filters and analysed colorimetrically on a segmented flow analyser (SAN++; Skalar).

Data analyses were conducted in R (R Core Team, 2022) on log_10_‐transformed data due to the spread of [BSi] among species. To test for differences between plant flower parts, we compared [BSi] using Wilcox tests, pairing samples from the same species. Variation in floral structure meant all parts could not be isolated in every species sampled, hence sample sizes differ among tests. All floral parts were compared with leaves of the same species also with paired Wilcox tests, which were also used to analyse the larger dataset, comparing whole flower and leaf [BSi]. To test for a relationship between whole flower and leaf [BSi], we used standardised major axis regression (Warton et al., 2012). Finally, analyses of variance, followed by Tukey post hoc analyses, were used to test for differences between [BSi] among plant groups, for both whole flowers and leaves.

To explore phylogenetic patterns, boxplots of leaf and whole flower [BSi] were prepared grouping plant orders and broader taxonomic groups. A phylogeny was generated by matching species names with the Open Tree of Life (online and updated, but see Hinchliff et al. (2015), a tree produced in January 2023) using the rotl package (Michonneau et al., 2016) and selecting the maximally resolved tree for the 104 species with both leaf and whole flower [BSi] (phylogenetic tree provided in Supporting Information). An analysis of traits (AOT) was carried out using the Phylocom package (Webb et al., 2008) to test whether divergences (*n* = 102) in leaf [BSi] were correlated with divergences in whole flower [BSi] throughout the phylogenetic tree. Pseudo‐branch lengths were not estimated, but were assigned a value of one as branch lengths are not required for this AOT. Analyses were performed on log_10_‐transformed data.

## 3 RESULTS

**FIGURE 1:**
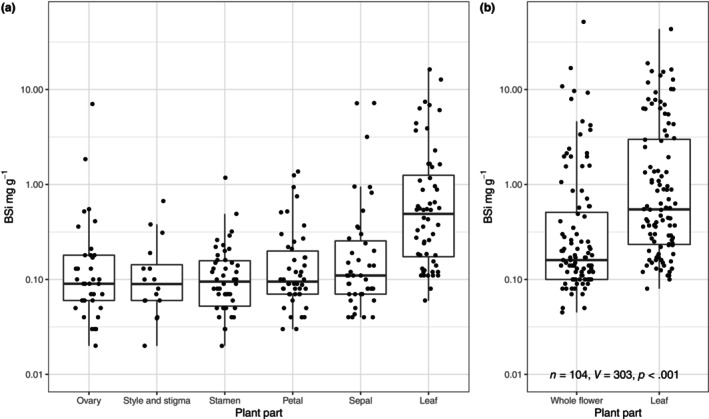
(a) [BSi] for different flower parts and leaves for 56 species. (b) [BSi] for whole flowers and leaves for 104 species. Note log scale of *y*‐axes. The values for flower [BSi] are overall higher in (b) because the larger dataset included more high Si accumulating species, and typically petals and sepals, which had (non‐significantly) higher [BSi], contributed a larger proportion of whole flower biomass than other plant parts shown in (a).

BSi was detected in all floral samples measured (Figure 1a,b). There was no difference in the [BSi] among floral parts within species, except sepals were marginally significantly higher than other floral parts (Figure 1a, Table 1). However, there was significantly less [BSi] in flower parts compared with leaves of the same species (Figure 1a, Table 1) and in whole flowers compared with leaves (Figure 1b, *V* = 303, *p* < .001, *n* = 104). The mean and standard error of whole flower [BSi] was 1.51 ± 0.55 mg g^−1^ and leaf [BSi] was 2.97 ± 0.57 mg g^−1^.

**TABLE 1:** Wilcox signed‐rank test results comparing [BSi] between plant parts (on paired samples) in Figure 1a.

	Leaf	Sepal	Petal	Stamen	Stigma and style	Ovary
Leaf		**<0.001**	**<0.001**	**<0.001**	**<0.001**	**<0.001**
Sepal	44		*0.013*	**<0.001**	*0.024*	*0.033*
Petal	47	41		0.137	0.638	0.927
Stamen	47	39	39		0.258	0.279
Stigma and style	17	14	15	16		0.451
Ovary	37	30	30	32	16	

*Note*: White squares report *p*‐values (significant results in bold, marginally significant results in italics), and grey boxes report numbers of species tested.

**FIGURE 2:**
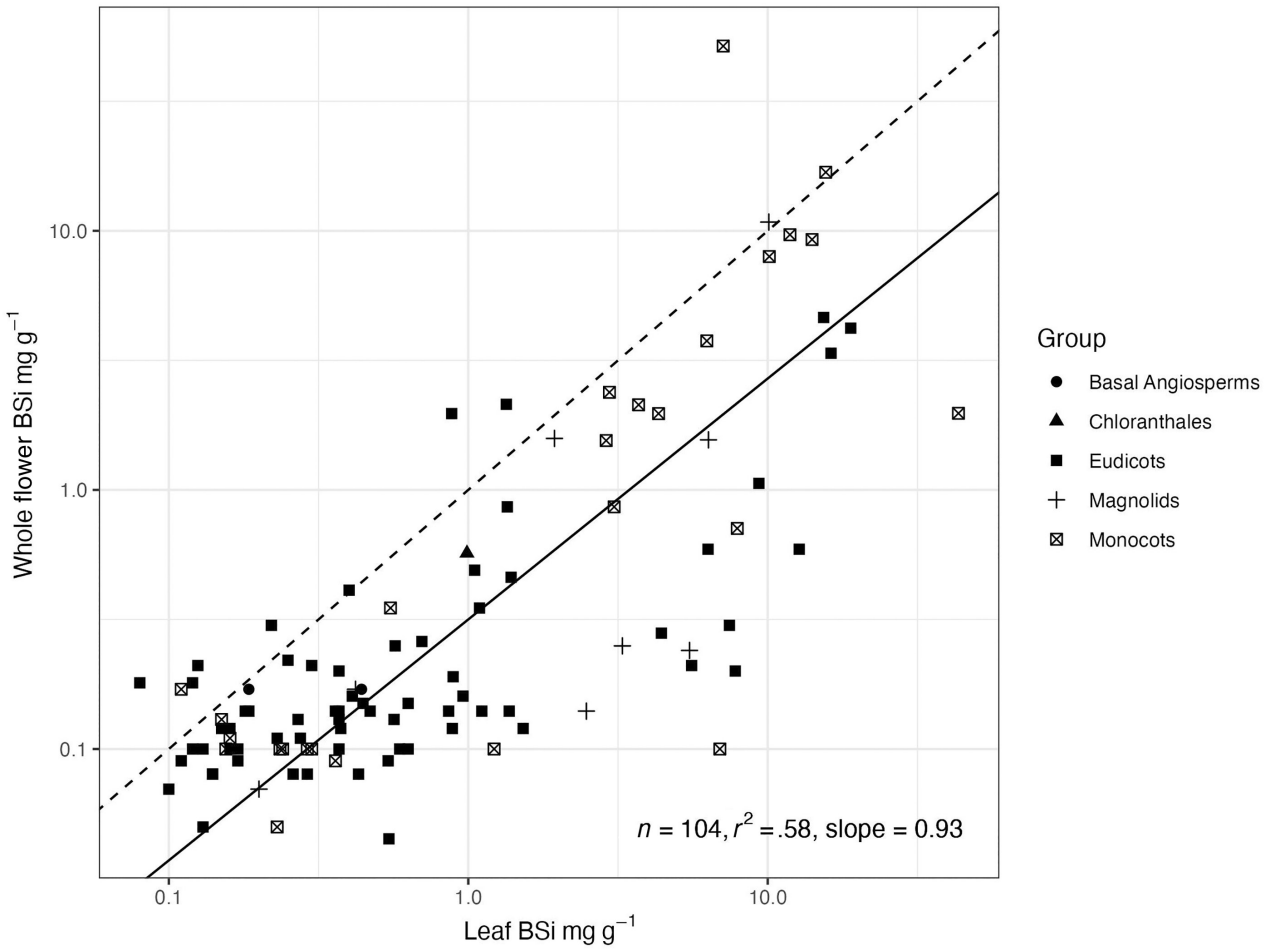
Comparisons between leaf and whole flower [BSi] by taxonomic group. The axes are log_10_ scale, with the regression line compared with a dotted 1:1 line.

There was a significant correlation between leaf and whole flower [BSi], with an *r* ^2^ of .58 (Figure 2). On average, whole flower [BSi] was 57% of leaf [BSi] concentration across 104 species, though eight species had higher [BSi] in flowers than in leaves, and three others are above but close to the 1:1 relationship (Figure 2). However, the slope of the correlation was .93 (Figure 2) indicating that as [BSi] in leaves increased, the [BSi] in flowers increased slightly less quickly.

**FIGURE 3:**
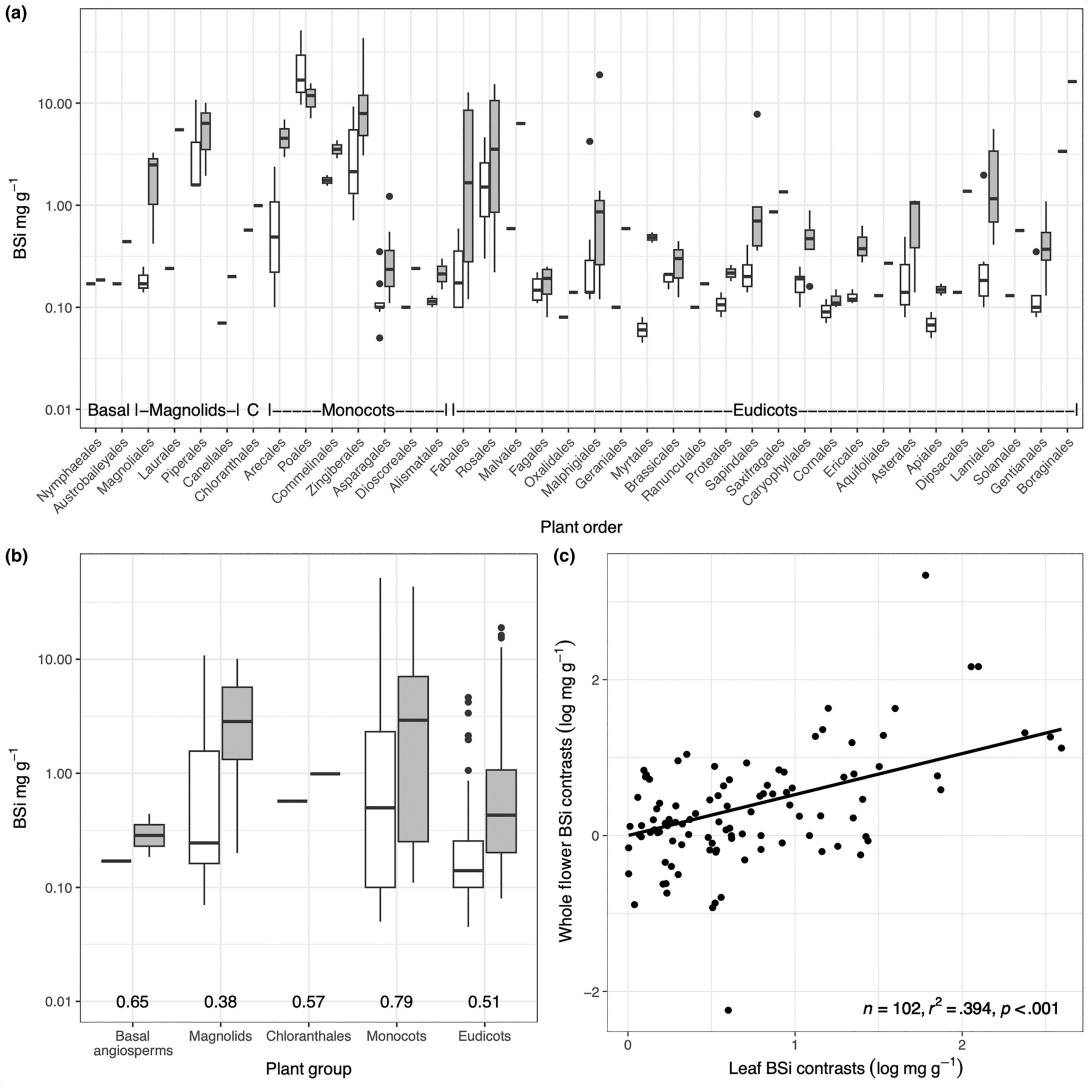
(a) Comparisons between whole flower (white) and leaf (grey) [BSi] by order, with plant groups indicated also (C = Chloranthales). The plant orders are arranged such that they align from L‐R with the phylogenetic tree in Figure S1 from top to bottom. (b) Comparisons between whole flower (white) and leaf (grey) [BSi] by plant group, with mean flower: leaf BSi ratios given. (c) Phylogenetically independent contrasts between leaf and whole flower [BSi] for 102 contrasts calculated at dichotomies across 104 species for logged data. The slope was forced through the origin as the sign value for each pair is arbitrary.

There was a significant difference between the [BSi] of whole flowers for the broader plant groups (df 4,99, *F* statistic = 3.97, *p* = .005), driven solely by a significant difference between Monocots and Eudicots (*p* = .002, Tukey multiple comparisons of means at a 95% group‐wise confidence level) due to the high [BSi] of Monocots compared with Eudicots (Figure 3b). The lack of significant differences between some other groups (especially Basal Angiosperms and Chloranthales) may be due to the small sample sizes. There was a marginally significant difference among groups in the [BSi] of leaves (df 4,99, *F* statistic = 3.07, *p* = .02), also driven by a significant difference between Monocots and Eudicots (*p* = .046).

In the basal groups of the Angiosperms, we observe on average a low [BSi] in both leaves and flowers (Figure 3a,b). In Magnoliids, we see a relatively high mean concentration, especially in the orders Laurales (in leaves) and Piperales (in flowers and leaves). Chlorantales, represented by one species, has a [BSi] intermediate to that of Magnoliids and Monocots and higher than that of Eudicots. On average, Monocots are similar to Magnoliids. The orders Arecales (in leaves), Poales, Commelinales and Zingiberales (all three in flowers and leaves) have a markedly higher BSi concentration than the other Monocot orders. These four orders together form the commelinid clade of Monocots. Eudicot [BSi] is generally lower than that of the other groups. But both orders with relatively low [BSi], like Apiales and Cornales, and orders with relatively higher BSi concentrations, like Fabales, Rosales, Malphigiales and Malvales, occur. These observed higher concentrations occur in different subclades within the Eudicots. The accumulation of BSi in leaves vs. flowers is not independent of phylogeny as there was a significant correlation among phylogenetically independent contrasts in the dataset (Figure 3c). Across most nodes in a maximally resolved tree of the species measured, a shift to higher leaf [BSi] was strongly associated with a shift to a higher flower [BSi].

## 4 DISCUSSION

### 4.1 Flowers are siliceous

Each species in this study contained floral Si with no differences among the various flower parts detected, except the sepals generally had intermediate [BSi] between other flower parts and leaves. There was however, much variation in [BSi] across the taxa examined, with some species accumulating very high [Si] (with 51.65 mg g^−1^ of dry weight in the Poales species *Cympogogon nardus*) and some largely excluding Si (<0.04 mg g^−1^ of dry weight in *Fuschia* sp. [Myrtales]). The variation among species generally mirrored the variation in leaf [BSi], and there was a phylogenetic correlation. This suggests that processes that drive uptake and precipitation of leaf [BSi] are also responsible for flower [BSi], which is in line with flowers being modified leaves.

Overall, leaf [BSi] was significantly higher than flower [BSi]. Photosynthesis can occur in both sterile and fertile parts of reproductive organs, especially when petals start green, and later become coloured, and sepals usually remain green and photosynthetic throughout a flower's lifespan (Aschan et al., 2005). Flowers have a cuticle to prevent water loss (Riederer & Muller, 2008), and stomata are present in the epidermis of petals and sepals, though generally less numerous than in leaves, and some are even non‐functional (Zhang et al., 2018; Ziegler, 1987). Flower longevity also varies between species, even in the same habitat, between male and female flowers of the same species, and across different pollination systems (Primack, 1985). Flower longevity depends on several factors, but is often much shorter than that of leaves, meaning less time for Si accumulation. Taken together, lower transpiration and shorter lifespan, in flowers vs. leaves, could explain why flower [Si] was generally about half of that in the leaves of the same plant, and why the correlation coefficient across species was lower than 1.

Roddy et al. (2016) investigated differences in flower water loss between clades of Angiosperms and showed that Monocots and Eudicots possess traits to limit water loss in their flowers. These features were not found in basal clades and Magnoliids where water loss along flowers was relatively high. Following Si uptake, if only transpiration and thus water loss were responsible for the [BSi] in the flowers, we would expect the flower‐to‐leaf [BSi] ratio to be higher in the basal Angiosperms and Magnoliids clade compared with the Monocots and Eudicots clades. As this is not what we have observed, notably with Magnoliids showing the lowest ratios, it seems unlikely that transpiration is the sole driver of Si accumulation in flowers. Moreover, in about 10% of results (11 species), floral [BSi] was equal or even higher than leaf [BSi]. These species belong to the Magnoliids (1 spp.), Monocots (3 spp.) and Eudicots (7 spp.) and may be good candidates for further study of BSi in flowers where there may be active Si accumulation involving as yet unidentified transporters and/or novel BSi functions.

### 4.2 Gene expression of Si transporters

The drivers of variation in Si accumulation in Angiosperms is a topic of active research. One explanation could be differences in transpiration, as described above. This would then be a purely passive consequence of selection at the root level, and of photosynthetic activity at flower level. Another explanation could be the presence of Si transporters, known to actively accumulate Si from the soil solution, and others directing Si to different plant parts in some species (reviewed in Mandlik et al., 2020). Transporters have been described in Monocot (Mitani & Ma, 2005) and Eudicot vegetative parts (Ma & Yamaji, 2015). In rice, a transporter (Lsi6) has been identified that redirects Si at nodes, and the expression of genes encoding the transporter is significantly increased in the node directly below the panicle when rice inflorescences (panicles) are completely emerged (Yamaji & Ma, 2009). Hence, the capacity to produce Si transporters is likely to be an important factor in determining which orders or families can engage in strong Si accumulation, and which cannot, including in their flowers, but to date, Si transporters are poorly studied beyond grasses.

Whalen et al. (2023) deduced from a study of phytolith production in a basal, morphologically conserved vascular plant species (*Lycopodiella alopecuroides*) that silica deposition initially occurred incidentally with passive uptake, and Si deposited at transpiration termini, and adaptations for functional silicon use by plants evolved subsequently. Trembath‐Reichert et al. (2015) examined [BSi] in extant members of early‐diverging plant clades. They concluded that silica biomineralisation is widespread across terrestrial plant lineages, but that the modified aquaporins responsible for Si transportation in angiosperms are not found within gymnosperms or in spore‐bearing plants, including plant lineages that are known to contain many weight‐per cent BSi (e.g. lycophytes and early‐diverging ferns). The most basal species of the Angiosperms is *Amborella trichopoda*, and as a sister group of all other Angiosperms would thus have first split off from the other Angiosperms before all other flowering plants did so. Ma and Yamaji (2015) found this species has genes similar to those that code for Si transporters in other species; however, in our results the basal Angiosperm species had very low [BSi]. Since the development of this type of Si channel appears to be unique to the Angiosperms (Ma & Yamaji, 2015), we concur to the reasoning of Trembath‐Reichert et al. (2015) that plants have a plastic capacity for silica accumulation, and this function has been gained and lost multiple times in the evolution of seed plants.

### 4.3 Phylogeny and pollination syndrome

Hodson et al. (2005) performed a meta‐analysis of [BSi] across taxonomically diverse foliar samples, covering a broader phylogenetic diversity (>600 angiosperms) than in this study. In general, they found ferns, Gymnosperms and Angiosperms accumulate less Si than non‐vascular plant species and horsetails (Equisetaceae), and Si accumulation was generally higher in Monocots than non‐Monocots. The latter difference was mostly driven by very high Si concentrations in the commelinid Monocots (orders Poales and Arecales; Hodson et al., 2005). Our measurements of floral (and leaf) BSi largely mirrored theirs.

Since BSi accumulation in flowers was observed across all species, it seems unlikely that pollinators suffer serious negative effects from Si in flower parts as there was not a strong shift towards flowers with very low or no [BSi] among any angiosperm group. However, in light of the evolution of land plants and silica biomineralisation (as outlined by Hodson et al., 2005; Ma & Yamaji, 2015; Trembath‐Reichert et al., 2015), there appears coincidence between the origin of flowers 180–140 million years ago (Bell et al., 2010), gains and losses of (functioning) genes, and overall less silica biomineralisation in comparison with more primitive plants. Our dataset is too limited to make hard claims, hence instead we discuss phylogeny in relation to pollination syndrome as a potential driver.

Basal angiosperms are mostly pollinated by ‘generalist’ insects that forage on phylogenetically unrelated plant species (Luo et al., 2018). Examples are flies, midges, thrips and beetles feeding on flower parts or ovipositing in flowers and their larvae feeding on flower parts (Luo & Zi, 1999). Flowers attract insects by their coloured parts, their odour and floral thermogenesis (heat production; Thien et al., 2009). Since pollinating insects or their larvae feed on all floral parts, including pollen, it seems logical that these flowers contain low [BSi]. Also, the most closely related clade to the Angiosperms, Gymnosperms, accumulate little Si (Hodson et al., 2005), which suggests that the ancestor of all current flowering plants was probably not a strong Si accumulator.

Magnoliids show the same generalist pollinator systems as the basal angiosperms with pollinator rewards being flower parts, hence the same low [BSi] is expected in the flower parts. The Magnoliids had intermediate‐to‐high [BSi] in flowers and leaves, with large variation, yet detailed information on pollinators at species level is lacking. The order of Canellales had a markedly lower [BSi] than the rest of the clade. The most parsimonious explanation is that the ancestral Magnoliid allowed Si accumulation in plant parts, but this property was secondarily lost in the order Canellales. Only the Piperales in this clade accumulate relatively high [BSi] in flowers. Within the genus *Piper*, there is still much debate about which pollination syndromes are important. However from several neotropical *Piper* species, it has been shown that insect pollination plays an important role (de Figueiredo & Sazima, 2004).

The Chlorantales seem to be a trichotomy with the Magnoliids and the Monocot‐Ceratophyllales‐Eudicot clade (Angiosperm Phylogeny Group (APG) et al., 2016) and can provide insight into the early diversification of angiosperms (Guo et al., 2021). This order has a higher [BSi] than in the basal Angiosperms and Magnoliids, especially in the flowers. Although represented by only one species in this study, it adds evidence to the hypothesis that the ability of the plant to accumulate Si was already present in the ancestral angiosperm species, but this function was gained and lost multiple times.

The Monocots are high Si accumulators (Hodson et al., 2005), especially the monophyletic commelinid clade which includes grasses (Poales). Grasses have a very different flower structure to other Angiosperms. In its typical form, a grass floret consists of an ovary surmounted by two styles with stigmas and three stamens and subtended by two small scales, called lodicules, considered to be remnants of the perianth. These parts are enfolded by two bract‐like structures, the lemma on the outside and the membranous palea on the inside. One or more florets (each with lemma and palea) are arranged in spikelets, each of which is subtended by two empty (sterile) glumes. The spikelets, in their turn, are arranged into inflorescences. In our analysis, we used the whole spikelet and did not separate glumes, lemma and palea from the ‘true’ flower parts (lodicules, stamens, styles and stigmas and ovary). This means that our BSi values for the Poales samples are not entirely congruent with the BSi values of species of the other plant groups since they include bract‐like parts (glumes, lemma and palea) that were not included in the other plant groups. However, the nature of the lemma and palea has long been debated. Historically, these outer structures of the grass floret were interpreted as bract and prophyll, hence non‐floral parts. However, recent molecular studies suggest they may be equivalent to sepal parts in other flowering plant groups (Lombardo & Yoshida, 2015). Therefore, it is only the inclusion of the two non‐fertile glumes in our flower samples that may have artificially increased our values for BSi in the flowers of the Poales (because of the high overall BSi level in the Poales). However, similar patterns of high floral BSi were also found in Commelinales and Zingiberales, which together with Poales belong to the commelinid clade of Monocots.

Within the commelinids, different pollination syndromes occur, and in Poales and Zingiberales, the ancestral pollination is animal‐mediated (Specht et al., 2012; Wolowski & Freitas, 2015). Wind pollination has evolved several times independently within the Angiosperms, and studies suggest that even within the Poales wind pollination has occurred independently five times (Givnish et al., 2010). Within the almost exclusively wind‐pollinated grasses (Poaceae) are examples of insect pollination (Adams et al., 1981). The [BSi] in commelinid Monocots is higher in both leaves and flowers than most non‐commelinid Monocots, especially in leaves. The most parsimonious explanation is that the ancestor of the commelinid Monocots developed strong Si accumulation. Whether this then drove wind pollination to arise independently in the different orders, as a better alternative to Si‐deterred insect pollinators, remains speculative at best.

BSi is infrequently studied in inflorescences, in comparison with leaves, but existing literature is associated with the Poales. Some pioneering work on understanding Si deposition in cell walls and lumen was carried out in *Phalaris* sp. lemmas and glumes respectively (Hodson et al., 1984, 1985). There is also significant research in Si accumulation in rice inflorescences such as investigations into genotypic variation of silica layer thickness in *Oryza sativa* lemmas, thought to limit cuticular transpiration, reduce water stress and improve flower fertility (Garrity et al., 1984). Kumar et al. (2017) record that prickles, macro‐hairs, long and short cells, papillae and stomata can be silicified in grass glumes and lemmas. Dendritic phytoliths, produced in grass inflorescences, are used to trace human use of cereals, including Panicoididae grasses in sub‐Saharan Africa (Novello & Barboni, 2015). Distinctive phytoliths have also been isolated from Bambusoideae inflorescences (Piperno & Pearsall, 1998). It is suggested that the abrasive bracts of *Setaria* sp. (Panicoideae) and *Phalaris* sp. (Pooidae) cause human oesophageal cancer given their high [BSi] in flowers and high cancer incidence where these species form a large dietary component (Parry & Hodson, 1982; Sangster et al., 1983). It could be that [BSi] protects grass inflorescences from herbivory, but perhaps also, or instead, the siliceous bracts protect the seeds inside them; Si accumulation potential in flowers is more to enable supply for seed development. In *Oryza sativa* (rice), silicification of the rachilla is associated with reduced panical shattering (grains remaining on the plant rather than being dispersed; Ge et al., 2022). In addition, Lindtner et al. (2021) suggest that silica hairs on the awns of wheat appendages are critical to locking the awns in place, contributing to the mechanism allowing seed movement along the soil.

Hodson et al. (2005) described low leaf [BSi] in Eudicot orders Brassicales, Aquifoliales, Cornales and Fabales. In our results, these orders also had among the lowest concentrations. Interestingly, up to seven Eudicot species had higher floral Si than leaf Si. Especially, high [BSi] was found in one species of the Boraginales. Our two representatives of the Boraginales are from the Boraginaceae (see Table S1), a family known to produce phytoliths (Wallis, 2003). The relationship between the Boraginales and other lamiids is not fully known (Angiosperm Phylogeny Group (APG) et al., 2016), making the Boraginales an interesting group to study, to find out why they accumulate more Si than other species in the Lamiid clade or the superasterid clade. In addition, Hodson et al. (2005) described a higher concentration in some species of the orders Fagales, Rosales, Asterales and Caryophyllales. This study also showed a slightly higher Si accumulation in both leaves and flowers in orders Fagales and Rosales than in other Eudicots. In Asterales and Caryophyllales, this is not seen in our results. In Eudicots, the most parsimonious explanation would therefore be that a mechanism that enables Si accumulation has arisen multiple times in different orders, maybe as a result of flower‐pollinator interactions. There seems no phylogenetic pattern among Eudicots in Si accumulation. Even within orders, there are differences between different families and even genera (Hodson et al., 2005; Honaine et al., 2021). Some orders such as Rosales, for example, include families with a very high phytolith production (Urticaceae) as well as very low (Rhamnaceae; Honaine et al., 2021). In these cases, Si accumulation has been gained or lost in specific families or genera.

### 4.4 Functions of Si in flowers

Given there is Si in flowers, what might its functions be? There are likely to be phylogenetic patterns to flower BSi functions, with grasses an example where high Si in flowers may have a protective function. Silica is purported to affect leaf colour, as structural colour (Strout et al., 2013), hence it could have a role in pollinator attraction through flower coloration. While over 85% of flowering plant species have evolved to attract and use animal pollinators (Ollerton et al., 2011), there are animal species which obtain nutritious pollen and energy‐rich nectar without pollinating flowers (floral larceny). Many such invertebrates penetrate the side of the flower to obtain nectar and pollen while avoiding the stigma and anthers (Irwin et al., 2001), or mammalian herbivores can consume whole flowers (e.g. Galetti & Pedroni, 1994), and floral BSi could be a defence against this very specific type of herbivory in a similar way to leaf antiherbivore defences. Species with shorter‐lived leaves accumulate more Si, and it has been hypothesised that these species use Si as a metabolically cheaper, but less versatile resource (Cooke & Leishman, 2011). As flowers are often short‐lived organs compared with others, plants could have evolved to use Si to support sepals and petals. Alternatively, Si facilitates the amelioration of a range of stresses (e.g. water and oxidative stress) and as flowers (through pollination) are generally critical to plant reproductive fitness, they could use Si to ameliorate the impacts of stresses incurred. The benefits of Si in stress alleviation are not limited to high Si accumulating species (Cooke & Leishman, 2016).

## 5 CONCLUSION

This study demonstrated that, across diverse taxa, flowers do accumulate BSi, with no significant variation of BSi among flower parts. Generally, floral BSi was lower than in leaves within the same species, but with some exceptions, and a phylogenetic correlation between floral and leaf BSi suggests that plant Si uptake mechanisms may drive accumulation rates in both organs. From aligning [BSi] in flowers to phylogenetic evolution of flowering plants, we conclude that floral BSi accumulation capacity evolved several times independently in the Magnoliids, commelinid Monocots and in some orders of the Eudicots. No clear phylogenetic trends were observed across Angiosperm plant groups: Both high and low floral [BSi] can be present. The genes that are responsible for Si transporters seem to co‐emerge with the evolution of flowers, though this remains very speculative. The commelinid Monocots clearly accumulate more Si relative to other related Monocots (both in leaves and flowers). In certain groups, such as grasses, this silification coincides with strong flower modifications, notably the reduction in the perianth and the modification of its parts into green, bract‐like structures and dominance of wind pollination.

While flowers generally have a lower [BSi] than leaves, it is not clear whether this results from a passive consequence of transpiration, or whether it is an active strategy. The capacity to accumulate BSi (both in leaves and flowers) appears multiple times in species across the whole phylogenetic variation. Contrary to our original hypothesis that BSi in flowers could be detrimental, and hence selected against, we found that BSi accumulation in flowers is widespread. Some flowers even have a higher [BSi] than their leaves and they show insect pollination (5 spp.), bird pollination (1 sp.) and wind pollination (5 spp.). Foliar BSi accumulation has been firmly incorporated into leaf trait frameworks (de Tombeur et al., 2022), and our findings suggest it would be promising to incorporate flower BSi into floral trait analyses, to consider the role of BSi in pollination syndromes and as defence against floral larceny and to explore potential contributions to plant reproductive fitness.

## AUTHOR CONTRIBUTIONS

**Jonas Schoelynck:** Conceptualization (lead); funding acquisition (lead); investigation (equal); project administration (lead); supervision (lead); writing – original draft (equal); writing – review and editing (equal). **Petra De Block:** Data curation (lead); investigation (equal); methodology (equal); validation (lead); writing – original draft (equal); writing – review and editing (equal). **Eva Van Dyck:** Data curation (lead); investigation (equal); writing – original draft (supporting); writing – review and editing (supporting). **Julia Cooke:** Formal analysis (equal); investigation (equal); software (lead); visualization (lead); writing – original draft (equal); writing – review and editing (equal).

## Supporting information


Figure S1.

Table S1.

Table S2.

## Data Availability

All data supporting the results are submitted in supplementary files to this manuscript (Tables S1 and S2).
