# Supplementary figures and images for: Is there silicon in flowers and what does it tell us?
Source: Ecol Evol. 2023 Oct 17;13(10):e10630. doi: 10.1002/ece3.10630 (PMC10580012; doi:10.1002/ece3.10630)

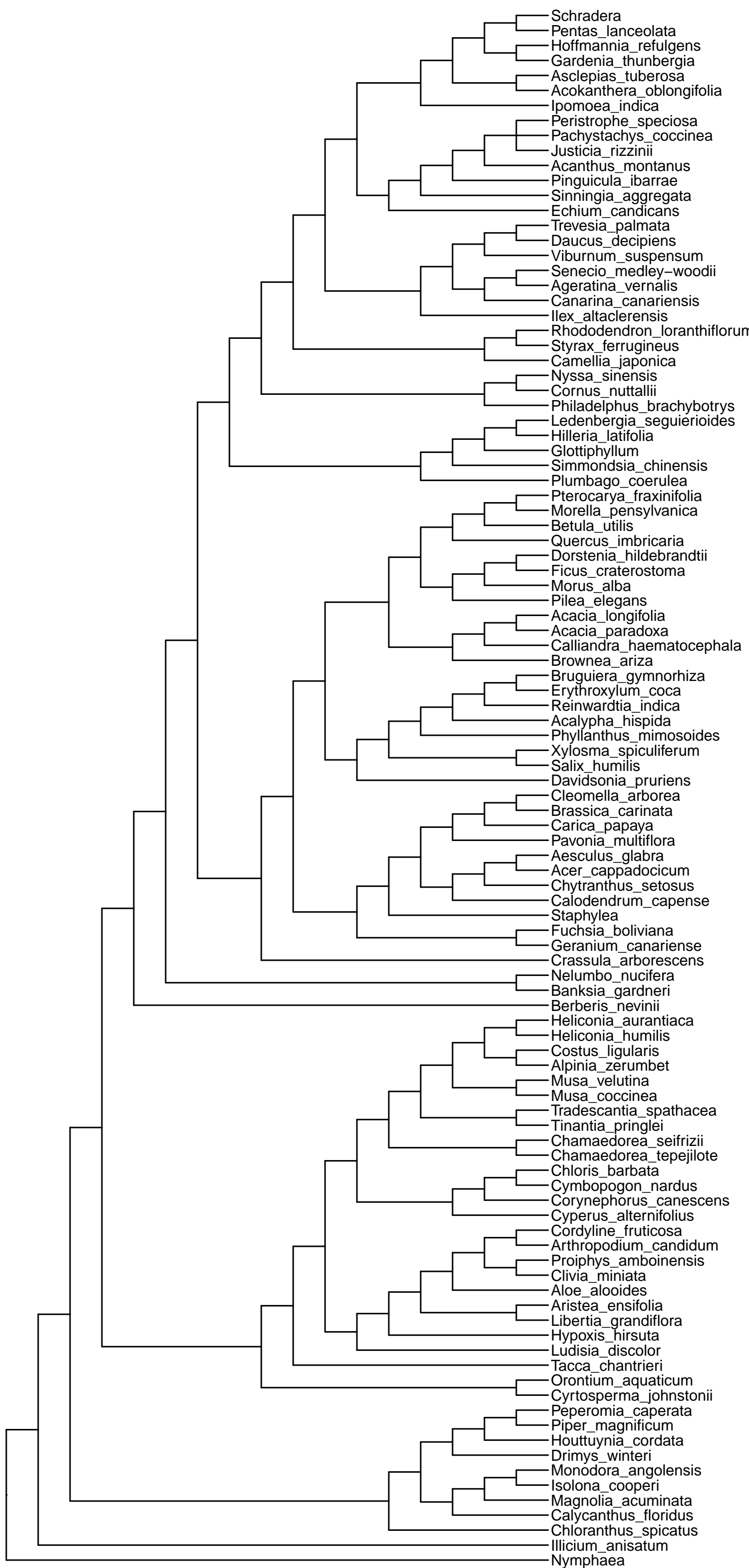

Supplement: Supplementary file 1 — Figure S1. [file ECE3-13-e10630-s002.pdf]
